# Integrating machine learning and statistical analysis to forecast insufficient physical activity trends using socio-demographic predictors

**DOI:** 10.3389/fpubh.2026.1776521

**Published:** 2026-06-16

**Authors:** Gelin Zhang, Lihua Yu

**Affiliations:** 1 School of Physical Education, Changsha University of Science and Technology, Changsha, Hunan, China; 2 School of Physical Education, Yanshan University, Qinhuangdao, Hebei, China

**Keywords:** global health, machine learning, physical activity, sex differences, statistical analysis

## Abstract

**Introduction:**

Insufficient physical activity is a major global health issue with disparities across sex and regions. This study examines these inequalities and projects future trends.

**Methods:**

World Health Organization data were analyzed using a Linear Mixed-Effects Model to assess the effects of sex, time, and region, accounting for country-level variation. Geospatial analysis was conducted, and four machine learning models (XGBoost, Random Forest, Multi-Layer Perceptron, Support Vector Regression) were used for forecasting.

**Results:**

Females showed higher and more variable levels of insufficient physical activity than males. Prevalence was lower in Africa and South-East Asia and higher in the Americas and Eastern Mediterranean. Trends worsened over time, particularly among females and high-prevalence regions. Forecasts differed: tree-based models suggested stabilization, while neural and kernel-based models projected continued increases.

**Discussion:**

Persistent disparities by sex and region remain. Variation across models highlights uncertainty in projections, supporting ensemble approaches and the need for targeted, gender-responsive interventions.

## 1 Introduction

Physical inactivity is one of the leading modifiable risk factors for non-communicable diseases globally. According to the World Health Organization (WHO) (26), approximately 1.8 billion adults worldwide do not meet recommended levels of physical activity, placing them at increased risk of cardiovascular disease, type 2 diabetes, cancer, and premature mortality. Marked disparities in physical inactivity exist across sexes and world regions: women consistently report higher prevalence of insufficient physical activity than men, and high-income regions such as the Americas and Europe show higher inactivity rates than lower-income regions such as Africa and South-East Asia (25). Understanding these disparities and forecasting their future trajectory is critical for evidence-based public health policy and resource allocation. One of the most relevant global development issues remains the disparity in the health and well-being of different populations. There are still gaps in progress regarding health, technology, and policies across sex, region, and socioeconomic status. Reports from the WHO (1) continue to capture the disproportionate health status and limited accessibility to vital health services among low- and middle-income populations relative to high-income populations. Furthermore, health outcomes continue to be shaped by sex differentials determined by biological and socio-cultural factors, as well as structural inequities within and across populations, leading to disparities in the incidence of diseases and the utilization of health services (2, 3). There is a need to conduct systematic analyses that are comprehensive in scope and predictive in approach (4, 5). Socio-demographic predictors including sex, WHO region, and country were incorporated as input features in all machine learning models used in this study, enabling sex-disaggregated and region-specific forecasts that go beyond simple time-series extrapolation.

The WHO Global Health Observatory offers a comprehensive and longitudinal repository of population-based health indicators across countries, regions, and demographic groups (6). These datasets enable systematic comparisons of health outcomes by sex and geography, and allow examination of trends over extended periods. However, reliance on descriptive statistics or regional averages alone can obscure substantial within-region and between-country variation. For example, while global averages may suggest gradual shifts in population health indicators, disaggregated analyses frequently reveal widening disparities, particularly with Africa and South-East Asia remaining consistently lower than regions such as the Americas and Europe (7). Likewise, persistent female–male differentials have been documented across diverse contexts, reflecting intersecting biological, social, and structural determinants of health (1, 2). Consequently, addressing these inequalities requires analytic approaches that extend beyond simple descriptive summaries, incorporating advanced statistical modeling capable of accounting for hierarchical data structures, contextual influences, and multilevel sources of variability. Traditional statistical methods such as ARIMA or linear regression, while useful for trend description, are limited in their ability to handle nonlinear relationships, interaction effects between socio-demographic variables, and hierarchical data structures inherent in multi-country, sex-disaggregated longitudinal datasets. Machine learning (ML) methods offer key advantages in this context: they learn adaptively from historical patterns, can model complex interactions among predictors, do not require distributional assumptions, and can generate stratified forecasts by sex and region simultaneously. At the same time, the growing availability of longitudinal health data raises important questions about forecasting. The objective of this study is to examine sex-based and regional disparities in the prevalence of insufficient physical activity using WHO longitudinal data (2000–2022), and to forecast future trends using four machine learning models (XGBoost, Random Forest, MLP, and SVR) with socio-demographic predictors (sex, WHO region, and country). Policymakers and public health stakeholders need reliable predictions to allocate resources, design interventions, and track progress toward goals such as the United Nations Sustainable Development Goals (SDGs) (5). Traditional time-series models, while useful, are limited in handling nonlinearities and complex interactions inherent in global health data. In contrast, machine learning (ML) offers a flexible and powerful framework for both pattern recognition and prediction (8, 9). Recent studies have demonstrated the utility of ML in public health, ranging from predicting disease outbreaks to forecasting healthcare demand (10, 11). Algorithms such as Random Forests and XGBoost provide robustness against noise and high dimensionality (12), while neural networks (e.g., Multi-Layer Perceptrons) excel at capturing nonlinear relationships (13). Similarly, Support Vector Regression (SVR), with its kernel-based approach, is adept at modeling complex nonlinear trends (14). Despite their proven success in other domains, the application of these methods to forecasting health disparities across sex and global regions remains limited. A systematic comparison of these algorithms within this context could therefore provide valuable insights into both methodological suitability and real-world forecasting reliability (15). Machine learning continues to demonstrate value outside of the health sciences as well. For instance, in materials science and manufacturing, ML is used to predict and optimize processing accuracy (16–19). It has also contributed to advances in fluid dynamics, materials discovery, and computational mechanics (20–22). These examples demonstrate the cross-disciplinary potential of ML and highlight its value not only in public health but also in engineering and applied sciences. Beyond health and engineering, machine learning has also had significant impacts on other scientific domains. In the life sciences and healthcare, ML has been applied to genomics, protein folding, and medical imaging, with applications continuing to expand (23). In physics, ML-driven approaches have supported quantum system analysis, cosmological simulation optimization, and particle physics data pattern recognition (24). Together, these examples illustrate the broad interdisciplinary relevance of machine learning and its transformative influence across both natural and applied sciences. This type of research contribute in several ways through the statistical rigor of predictive modeling. First, it documents and quantifies enduring disparities across sex and global health outcomes (6, 7), which reflect underlying structural inequalities. Second, it outlines temporal trajectories that provide evidence of both progress and stagnation across diverse societal contexts (3). Third, it demonstrates the utility of machine learning for forecasting health inequities, considering future applications in public health research (10, 11). Overall, these contributions offer a nuanced and predictive outlook on global and local contexts, yielding implications for future interventions and policymaking while enabling readers to directly address both current and emerging inequalities.

This study integrates statistical analysis, advanced data visualization, and machine learning forecasting to comprehensively examine health disparities across sex, region, and time. Our approach proceeds in three stages:

1. Descriptive and inferential statistics are applied to summarize patterns and test differences across sex and region.
2. Data visualization using boxplots, density plots, scatterplots, violin/strip plots, histograms, and heatmaps is employed to highlight disparities and trends in an accessible and interpretable manner.
3. Machine learning models (XGBoost, Random Forest, MLP, SVR) are implemented to forecast trajectories for the next five years, with comparisons of predictive accuracy, uncertainty, and trend behavior.

## 2 Methodology

### 2.1 Data source and preparation

Insufficient physical activity is defined according to WHO global recommendations as engaging in less than 150 min of moderate-intensity aerobic physical activity per week, or less than 75 min of vigorous-intensity aerobic physical activity per week, or an equivalent combination thereof (27). The prevalence estimates represent age-standardized proportions of adults aged 18 years and above who do not meet these recommendations, expressed as a percentage (%).

The data for this analysis were obtained from the World Health Organization (WHO) and consist of observations categorized by region (parent location), country or sub-location (Location), sex, and period (year). *Prevalence of Insufficient Physical Activity (%)* represents the health indicator or main outcome of interest. The dataset was organized and cleaned in preparation for analysis. Data cleaning included standardizing column names, ensuring consistent variable data types (categorical encoding for sex, Parent Location, and Location; numeric for period), and addressing missing values and inconsistencies. The processed dataset provided the basis for statistical modeling. All data preparation was conducted in Python using the pandas library.

The specific indicator used is “Prevalence of insufficient physical activity among adults aged 18 + years (age-standardized estimate) (%)” (WHO Global Health Observatory; GHO indicator code: NCD_PAC). The dataset spans 2000 to 2022 (23 years of longitudinal data) and covers 195 countries and territories across six WHO regions. Data are disaggregated by sex (Male, Female, and Both sexes), yielding 13,455 country-year-sex observations in total. No missing values were present in the outcome variable. The complete Python analysis code is available upon reasonable request from the corresponding author.

### 2.2 Statistical analysis

A Linear Mixed-Effects Model (LMM) was used to examine the effects of sex, time (period), and region (parent location) on the outcome *Prevalence of Insufficient Physical Activity (%).* This approach was appropriate because our dataset included multiple measures within the same Location, which could introduce correlation between observations. Conventional regression models assume independence among observations, which may lead to underestimation of variability and biased inference. The LMM allows variability to be decomposed into two sources:

- Fixed effects: systematic effects of interest (Sex, Period, ParentLocation).
- Random effects: unobserved heterogeneity across locations, modeled as random intercepts to account for overall baselines within a given location.

#### 2.2.1 Model specification

The general form of the model was:


$$\begin{array}{l} \mathrm{Yij}=\beta 0+\beta 1\;(\text{Sexij}\;)+\beta 2\;(\text{Periodij}\;)+\beta 3\;(\text{ParentLocationij}\;)\\ +\mathrm{uj}+\mathrm{\varepsilon ij}\; \end{array}$$ (1)

where:

*Yij*: Prevalence of Insufficient Physical Activity (%) for observation i in location *j*, *β₀*: Overall intercept, *β₁, β₂, β₃:* Fixed effect coefficients (sex, period, region), *uj:* Random effect for location _j_, assumed *uj ~ N(0, σ^2^u), εij*: Residual error, assumed *εij ~ N(0, σ^2^).*

This model structure allows for both population-level inference (fixed effects) and location-specific variability (random effects).

### 2.3 Outputs and interpretation

Model results are reported as regression coefficients, standard errors, z-values, *p*-values, and 95% confidence intervals. Predictors with *p* < 0.05 were considered statistically significant. Additionally, descriptive statistics were calculated by sex and region. Data patterns and distributions were examined using visualizations generated in Python (matplotlib and seaborn libraries), including boxplots, violin plots, scatterplots, density plots, and heatmaps.

## 3 Machine learning models

This section discusses the machine learning methods employed for prediction and forecasting. A total of four models were applied: XGBoost, Multi-Layer Perceptron (MLP), Random Forest (RF), and Support Vector Regression (SVR). Each method relies on distinct mechanisms and mathematical formulations, which are explained in the following section.

Four input features were used for all models: (1) Period (year, 2000–2022), (2) Sex (Male/Female, label-encoded), (3) WHO Region (6 categories, label-encoded), and (4) Country (195 categories, label-encoded). The outcome variable was log-transformed prior to training and back-transformed for evaluation. A temporal train/test split was applied, with 80% of data (2000–2018) used for training and 20% (2019–2022) for testing, ensuring chronological ordering to prevent data leakage. Table 1 summarises the hyperparameter configuration for each model. Forecast uncertainty is quantified using bootstrap-based 95% prediction intervals (1,000 bootstrap iterations).

**Table 1:** Hyperparameter configurations for all four machine learning models.

Model	Key hyperparameters
XGBoost	n_estimators = 1,000 | learning_rate = 0.05 | max_depth = 8 | subsample = 0.8 | colsample_bytree = 0.8 | random_state = 42
Random forest	n_estimators = 500 | max_depth = None | max_features = sqrt | random_state = 42
MLP (neural network)	hidden_layer_sizes = (100, 50) | activation = relu | solver = adam | learning_rate = 0.001 | max_iter = 500 | random_state = 42
SVR	kernel = rbf | C = 10 | epsilon = 0.1 | gamma = scale

### 3.1 XGBoost (extreme gradient boosting)

XGBoost is an optimized gradient boosting algorithm where an ensemble of weak learners (decision trees) is built consecutively. Each tree sequentially corrects the residual errors of the preceding decision tree by optimizing a loss function. The algorithm is in demand because it can handle large datasets while producing predictive models efficiently.

#### 3.1.1 Mathematical formulation

1 Objective function:

$$L(\varphi )=\Sigma \;l(\mathrm{yi},\hat{y}i)+\Sigma \;\Omega (\mathrm{fk})$$ (2)

where *l(y_i_, ŷ_i_)* is the loss function, and *Ω(fk)* is the regularization term.

2 Prediction: *ŷ_i_ = Σ f_k_(x_i_), f_k_ ∈ F* (set of regression trees)

### 3.2 Multi-layer perceptron (MLP)

The Multi-Layer Perceptron is a variety of feedforward artificial neural network, consisting of an input layer, two hidden layers (100 and 50 neurons respectively), and an output layer. The hidden layers apply a weighted linear transformation followed by a nonlinear activation function; the output layer uses a linear (identity) activation, as is standard for regression tasks. MLP is particularly suited to capturing complex nonlinear relationships and can, unlike tree-based models, extrapolate beyond the range of training data, which enables steeper projected trends.

#### 3.2.1 Mathematical formulation

1 Neuron Output:

hj=σ(Σwijxi+bj) (3)

where *σ* is the activation function, w_ij_ are weights, and b_j_ is the bias.

2 Prediction: *ŷ = Σ v_j_hj + c*

### 3.3 Random forest (RF)

Random Forest is an ensemble method that builds multiple decision trees using bootstrapped samples of the dataset and random subsets of features at each split. Predictions are obtained by averaging the outputs of all trees, which reduces variance and improves generalization.

#### 3.3.1 Mathematical formulation

1 Prediction:

$$\hat{y}=(1/T)\;\sum_{t=1}^{t}\mathrm{ht}(x),\text{for}\;t=1\;\text{to}\;T$$ (4)

where *h_t_(x)* is the prediction of the *t^th^* decision tree.

2 Ensemble: predictions are aggregated by averaging the outputs of all trees (mean prediction), which reduces variance and improves generalization.

### 3.4 Support vector regression (SVR)

Support Vector Regression is based on Support Vector Machines and aims to find a function that approximates data within a margin of tolerance (*ε*-insensitive zone). It minimizes prediction error while keeping the model complexity low, making it robust to outliers.

#### 3.4.1 Mathematical formulation

1 Optimization problem:

$$\mathrm{Minimize}\;(1/2){\left\|w\right\|}^{2}\;\text{subject to}\;|\mathrm{yi}-(w\cdot \mathrm{xi}+b)|\;\leq \varepsilon$$ (5)

2 Prediction Function: *ŷ* = *Σ*(*α*_*ᵢ*_ − α_ᵢ_*) *K*(*x_ᵢ_, x*) + *b*, *where K*(*x_ᵢ_, x*) = *exp*.(−*γ*‖*x_ᵢ_* − *x*‖^2^) *is the RBF kernel function*

**Figure 1:**
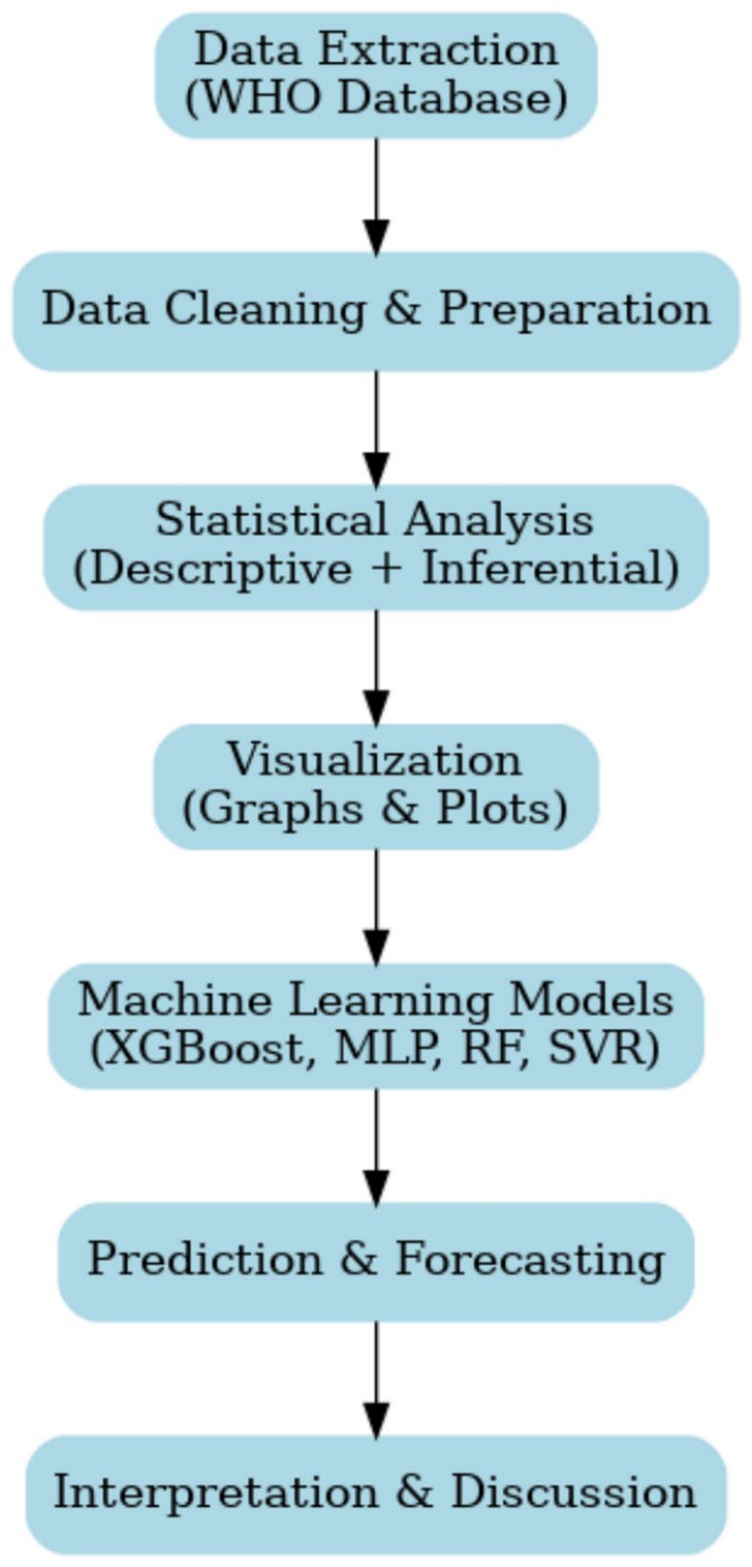
Flow chart of current study.

Figure 1 shows the flow chart of current study.

## 4 Result and discussion

The results of the Linear Mixed-Effects Model are presented in Table 2. Reported values include regression coefficients (*β*), standard errors, z-values, *p*-values, and 95% confidence intervals. The model intercept was negative and highly significant (β = −148.30, SE = 10.64, z = −13.94, *p* < 0.001), providing the baseline for interpreting the effects of sex, region, and time. Sex was a strong and consistent predictor. Female values were significantly higher (*β* = 3.97, SE = 0.09, z = 46.57, *p* < 0.001), while male values were significantly lower (β = −3.99, SE = 0.09, z = −46.84, *p* < 0.001), confirming persistent sex-based disparities, with women exhibiting greater levels of insufficient physical activity. Regional effects were also substantial. Using Africa as the reference category, insufficient physical activity was significantly higher in the Americas (β = 15.19, SE = 2.04, *p* < 0.001), the Eastern Mediterranean (β = 16.58, SE = 2.37, *p* < 0.001), Europe (β = 9.52, SE = 1.85, *p* < 0.001), and the Western Pacific (β = 8.52, SE = 2.22, *p* < 0.001). South-East Asia did not differ significantly from Africa (β = 2.87, SE = 3.08, *p* = 0.350). These effects indicate marked regional inequalities, with several regions consistently exhibiting higher inactivity levels, while others remain comparatively lower. Time (period) demonstrated a small but statistically significant positive effect (β = 0.08, SE = 0.01, z = 15.72, *p* < 0.001), indicating a gradual worsening in insufficient physical activity over time, rather than improvement. The random-effects variance at the location level (Var = 5.17, SE = 0.54, z = 9.63, *p* < 0.001) shows meaningful unexplained heterogeneity among countries, beyond the fixed effects of sex, region, and time. Together, these findings demonstrate persistent sex- and region-based disparities, a slowly worsening global trend, and the necessity of modeling location-specific variation in population physical activity.

**Table 2:** Results from the linear mixed-effects model (LMM) examining the effects of sex, region, and period on health outcomes [prevalence of insufficient physical activity (%)].

Predictor	Coefficient (*β*)	Std. error	z-value	*p*-value	95% CI lower	95% CI upper
Intercept	−148.30	10.64	−13.94	< 0.001	−169.15	−127.45
Sex (female)	3.97	0.09	46.57	< 0.001	3.80	4.14
Sex (male)	−3.99	0.09	−46.84	< 0.001	−4.16	−3.83
ParentLocation (Americas)	15.19	2.04	7.46	< 0.001	11.20	19.18
ParentLocation (Eastern Mediterranean)	16.58	2.37	6.98	< 0.001	11.93	21.23
ParentLocation (Europe)	9.52	1.85	5.15	< 0.001	5.90	13.15
ParentLocation (South-East Asia)	2.87	3.08	0.93	0.350	−3.16	8.91
ParentLocation (Western Pacific)	8.52	2.22	3.84	< 0.001	4.17	12.87
Period	0.08	0.01	15.72	< 0.001	0.07	0.09
Group variance	5.17	0.54	9.63	< 0.001	4.12	6.22

**Figure 2:**
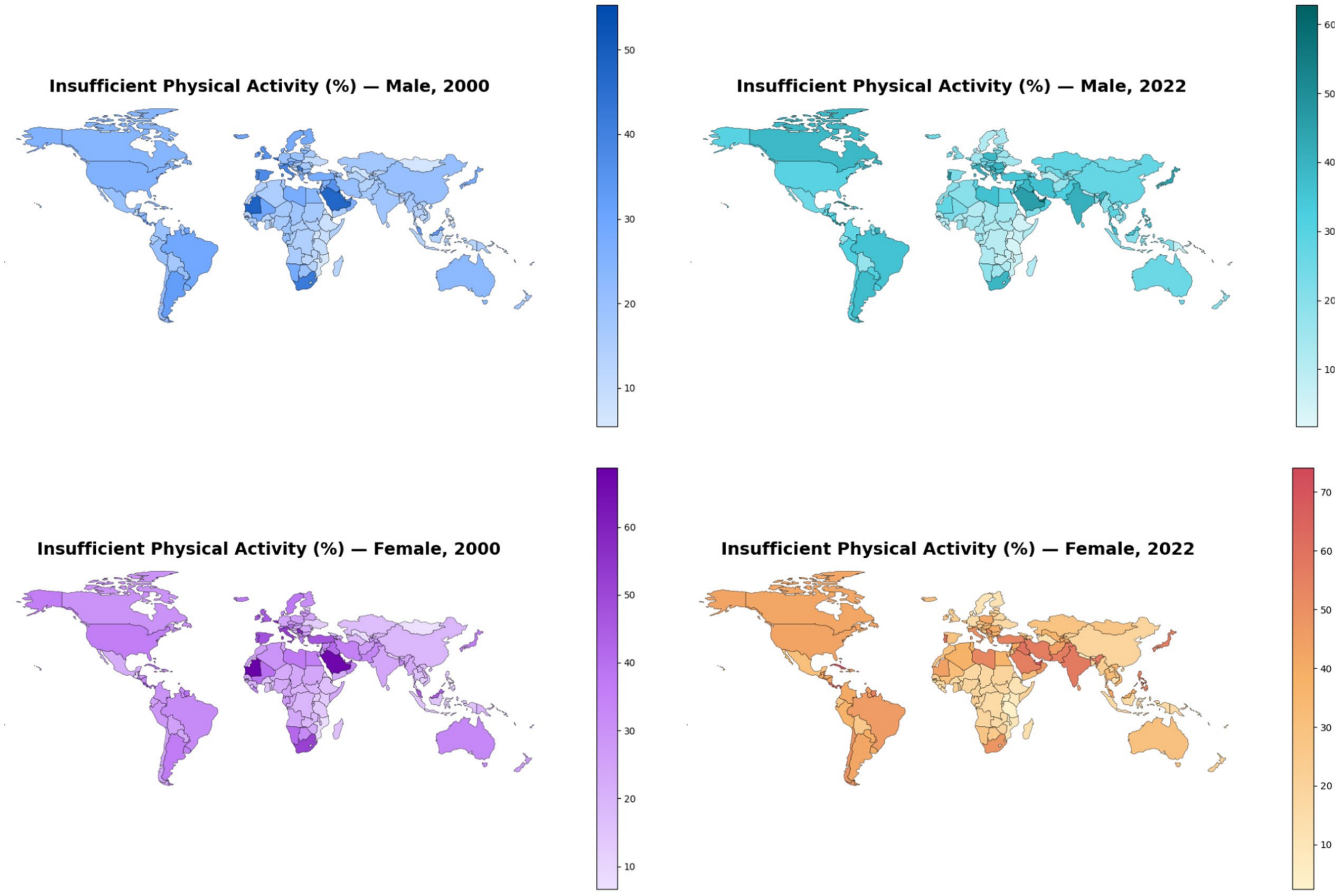
Choropleth maps of insufficient physical activity of males and females for 2000 and 2022.

The maps illustrate clear geographical and sex-related differences in insufficient physical activity across the two time points. In 2000, insufficient activity among males was relatively lower in many regions, whereas females showed higher inactivity levels, particularly in Middle Eastern and North African countries, parts of South Asia, and several high-income settings. Sub-Saharan African and some Southeast Asian countries displayed comparatively lower inactivity in both sexes during this period. By 2022, these patterns persisted and became more pronounced. Female inactivity remained highest in the Eastern Mediterranean region, where several countries exceeded 50–60%, while male inactivity also increased but to a lesser extent, maintaining a consistent sex gap. In many high-income countries in North America, Europe, and Oceania, inactivity rose in both sexes, reflecting lifestyle changes associated with urbanization and sedentary work. Meanwhile, physical inactivity levels in several lower-income regions remained relatively lower (indicating greater physical activity), although gradual increases in inactivity were also evident over time. The maps therefore depict a stable geographic pattern, with marked sex differences, particularly in regions where cultural, environmental, or social constraints may limit women’s opportunities for physical activity. Figure 2 shows the choropleth maps among the male and females.

The colour scale for both choropleth maps in Figure 2 has been unified to 0–75% across the 2000 and 2022 panels, enabling direct visual comparison of the change in prevalence of insufficient physical activity over time without visual bias.

**Figure 3:**
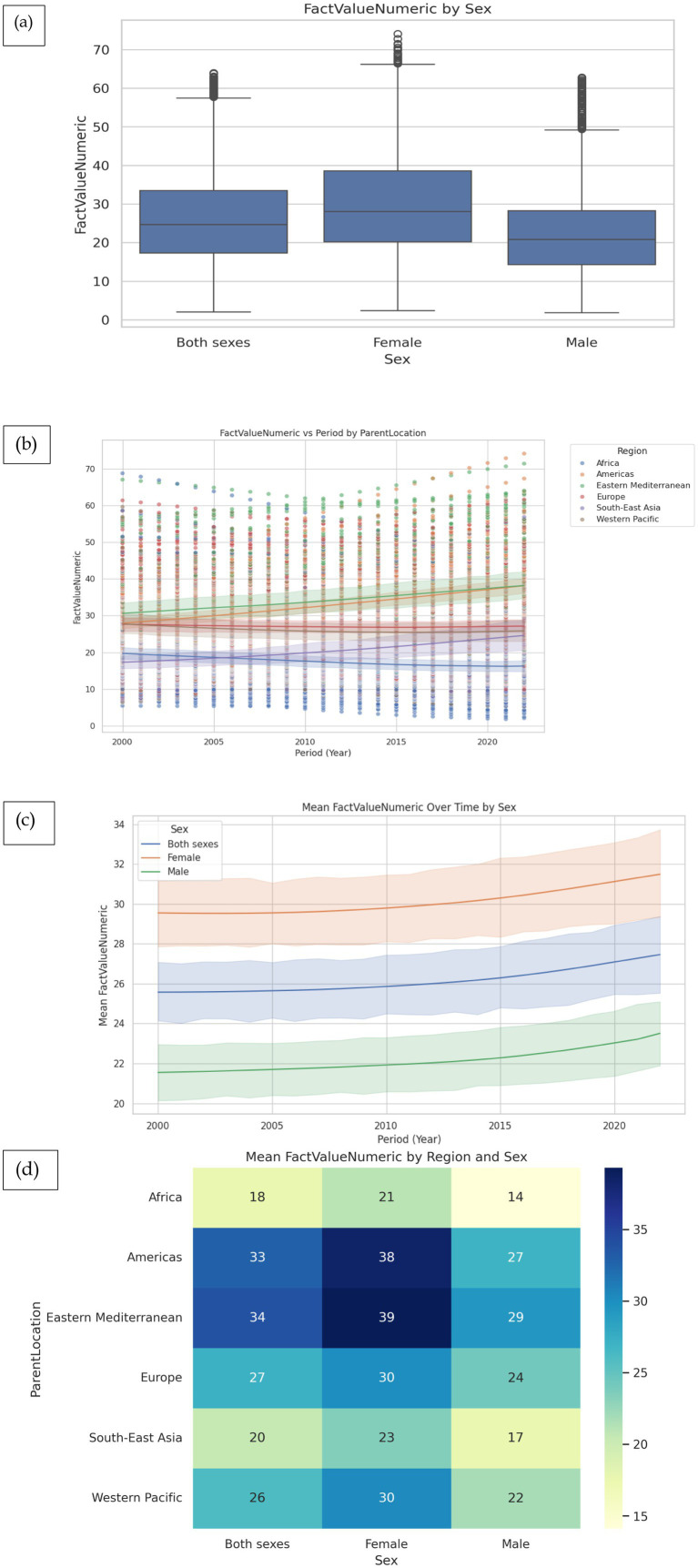
**(a)** Box plot of the achieved results, **(b)** the scatter plot of achieved results, **(c)** time-series graph, **(d)** heat map on the achieved results.

Figure 3a, demonstrates a clear and persistent sex-based disparity in insufficient physical activity. The distribution among females exhibits both a higher median and wider interquartile range compared to males, indicating that women not only experience higher average levels of inactivity, but also greater variability across populations. This wider spread suggests substantial heterogeneity in the social, cultural, and environmental constraints that shape women’s opportunities for physical activity. In many settings, particularly those characterized by restrictive gender norms, unequal domestic labor burdens, and limited access to safe public spaces, women may face structural barriers that do not equally affect men. The comparatively narrower distribution in male values reflects more consistent access to activity-supportive environments, such as participation in the workforce, commuting that incorporates mobility, and greater social acceptability of leisure-time physical activity. The persistence of this gap aligns with global surveillance findings reported in WHO STEPwise and GAPPA monitoring frameworks, underscoring that gender inequity remains a central determinant of physical inactivity worldwide.

Figure 3b, highlights broad geographic divergence in insufficient physical activity across global regions. Africa and South-East Asia maintain relatively lower prevalence levels over time, although with signs of gradual increases. These regions have historically sustained higher background levels of occupation-based and transportation-related physical activity due to lower mechanization, lower car ownership, and greater reliance on manual labor and active mobility. By contrast, the Americas and Eastern Mediterranean regions display both higher baseline inactivity and steeper upward trajectories, reflecting intensified urbanization, widespread sedentary occupations, greater dependence on automobiles, and fewer culturally or infrastructurally supported opportunities for recreational walking or sport participation. Europe and the Western Pacific occupy intermediate positions, showing moderate but steady increases, consistent with progressive lifestyle transitions and aging demographics.

The widening separation between regional trajectories indicates that global physical inactivity is not converging, but instead diverging along socioeconomic, cultural, and infrastructural lines. This reinforces that achievement of the WHO Global Action Plan on Physical Activity (GAPPA) 2018–2030 will require region-specific rather than universalized strategies, particularly in regions where structural barriers to active living are entrenched.

Figure 3c, presents the mean prevalence of insufficient physical activity among males and females over the study period, revealing parallel upward trends for both sexes. However, at every measured time point, the mean female prevalence exceeds the male mean, confirming that the sex difference observed in Figure 2a is not limited to specific contexts, but persistently sustained across decades. The parallel slopes suggest that the underlying drivers of increasing physical inactivity, such as urban densification without pedestrian-friendly design, expanded sedentary service economies, and increased screen-based leisure, affect both sexes globally. However, the consistently higher values among women indicate that interventions that increase opportunity for routine movement have disproportionately excluded women, either through limited access, safety constraints, cultural restrictions, or lack of targeted policy action.

The stability of this sex gap across time indicates that the disparity is structural rather than episodic. This finding directly challenges the feasibility of reaching SDG 3.4.1 (reduction of premature mortality through NCD prevention) without integrating gender-responsive physical activity promotion into health policy, education systems, urban design, and community sports infrastructure.

Figure 3d, across sex and area visually emphasizes differences. Particularly among women, the Eastern Mediterranean and Americas have the highest averages; Africa always shows the lowest throughout all sexes. Scientifically, this representation emphasizes the combined effects of geography and sex in determining results, with policy and infrastructure most probably playing major roles. The gradient throughout areas gives a quick visual verification of continuous worldwide injustices.

### 4.1 Machine learning models for forecasting

Table 3 shows the Performance metrics for all four ML models on the hold-out test set (2019–2022). All metrics computed on back-transformed predictions. Random Forest achieved the best fit (R^2^ = 0.9310); MLP and SVR showed substantially lower accuracy (R^2^ < 0.44).

**Table 3:** Performance metrics of all methods.

Model	R^2^	RMSE	MAE	MAPE (%)
XGBoost	0.9200	3.9248	2.8393	11.76%
Random forest	0.9310	3.6459	2.6318	10.73%
MLP (neural network)	0.4334	10.4455	7.6974	38.28%
SVR	0.4151	10.6128	7.7695	40.09%

**Figure 4:**
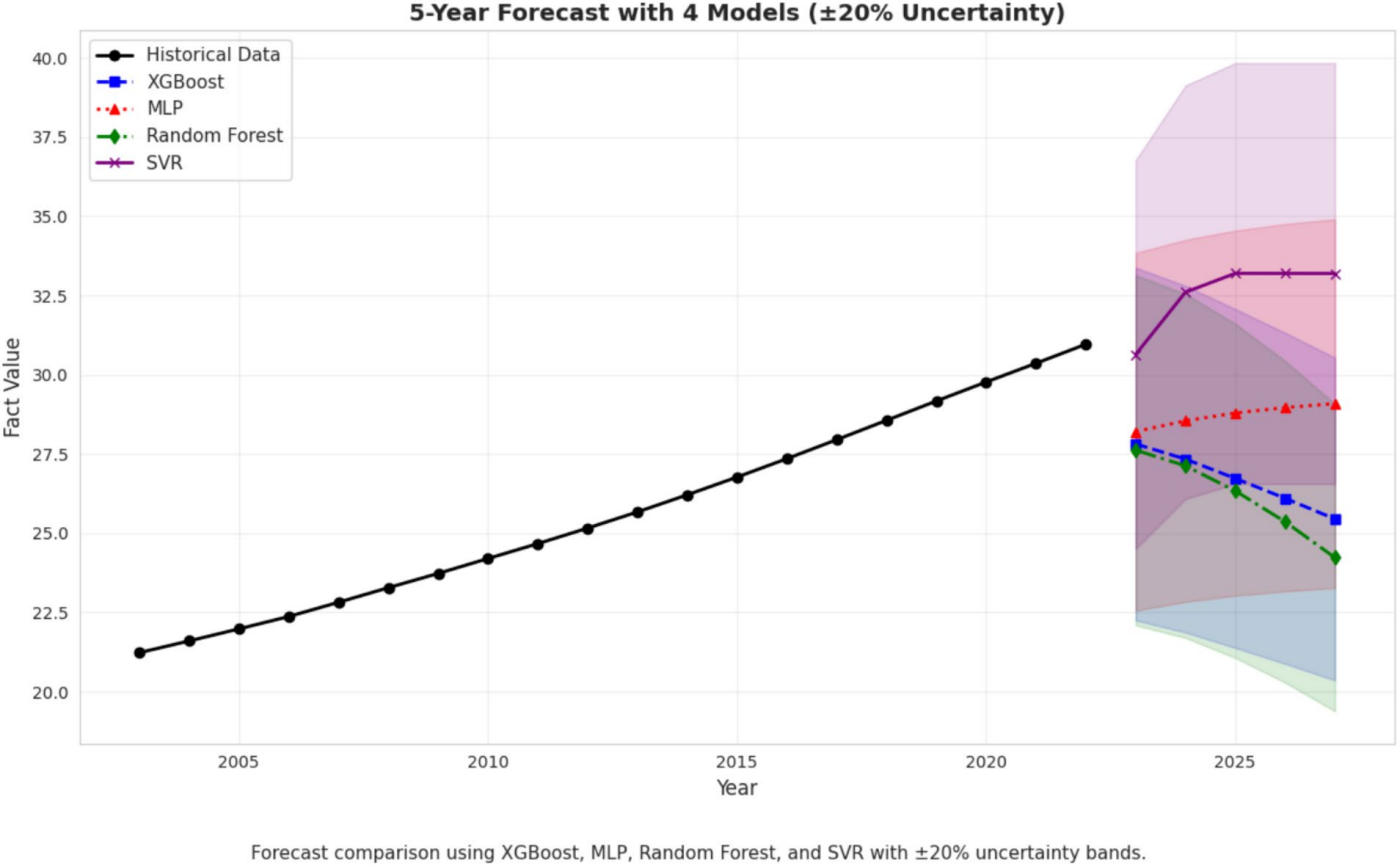
5-year forecast with XGBoost, random forest, MLP, and SVR models, with 95% bootstrap prediction intervals (faceted 2 × 2 layout, one panel per model).

Machine learning (ML) provides a flexible predictive framework because it can model nonlinear relationships and interaction effects without requiring strict statistical assumptions. Unlike conventional time-series approaches (e.g., ARIMA or exponential smoothing), ML methods learn adaptively from historical patterns and can capture complex multi-factor dynamics influencing population-level indicators. In this study, four ML algorithms: XGBoost, Random Forest, Multi-Layer Perceptron (MLP), and Support Vector Regression (SVR) were applied to forecast insufficient physical activity prevalence over the next five years Figure 4. These models represent different learning philosophies and therefore produce distinct projection profiles, underscoring the importance of model interpretability in public health forecasting.

XGBoost, a gradient-boosted ensemble of decision trees, performed well for short-term pattern fitting; however, its forecast showed stabilisation with a slight downward tendency after 2022. This likely reflects the model’s emphasis on minimizing recent residual error, thereby overweighting short-term fluctuations and underestimating longer-term upward momentum. Similarly, Random Forest, which aggregates predictions from many independent decision trees, projected stabilization followed by a modest decline. Such behavior reflects the model’s intrinsic tendency to regress toward the mean, producing stable but conservative forecasts that may understate future escalation.

In contrast, the MLP neural network captured ongoing upward momentum, projecting a steady rise consistent with long-term historical trends, reaching approximately 29% by 2026. SVR, which maps data into a higher-dimensional feature space using kernel transformations, produced the steepest projected increase (up to ~33–34%), alongside the widest uncertainty range. This suggests strong sensitivity to trend accelerations but also to parameter tuning and data variability.

Taken together, these patterns indicate that tree-based models yield conservative forecasts that dampen long-term trend continuation, whereas neural and kernel-based models allow steeper upward projections, implying greater potential worsening of inactivity levels. The divergence between models emphasizes that forecasts are model-dependent and should not be interpreted deterministically. Rather, they represent plausible trajectories conditioned on prior data behavior.

The divergence in model forecasts reflects fundamental architectural differences. Tree-based models (Random Forest, XGBoost) cannot extrapolate beyond the value range observed during training, as predictions are derived by averaging leaf-node values, causing forecasts to plateau near the last observed values. In contrast, MLP and SVR extrapolate beyond the training range via continuous functional transformations, yielding steeper upward trajectories. However, the hold-out validation results strongly favour tree-based models: Random Forest achieved R^2^ = 0.9310 and XGBoost R^2^ = 0.9200 on the test set (2019–2022), whereas MLP achieved only R^2^ = 0.4334 and SVR R^2^ = 0.4151. The steep upward forecasts of MLP and SVR are therefore not well-supported by their out-of-sample predictive performance and should be interpreted as upper-bound sensitivity scenarios. Given documented global trends in urbanisation, sedentary occupations, increased screen time, and post-COVID-19 behavioral changes (25, 26), the moderate upward trajectories projected by Random Forest and XGBoost are statistically credible and epidemiologically plausible. We recommend treating tree-based forecasts as primary estimates and adopting ensemble approaches in future work.

Therefore, forecast interpretation should be grounded not only in statistical prediction but also in contextual knowledge such as policy reforms, socioeconomic disruption, and global urbanization trends, which influence physical activity environments. The projected 95% bootstrap prediction intervals highlight that these forecasts represent scenarios, not fixed outcomes. Consistent with best practices in population health modeling, the findings support the use of ensemble or consensus forecasting to balance optimism and conservatism, reduce model bias, and improve decision relevance for policy planning.

## 5 Practical implications

The findings of this study hold significant practical value for public health stakeholders, policymakers, and researchers. By identifying sex-based and regional disparities and combining them with machine learning forecasts, the results provide actionable insights for both immediate interventions and long-term planning.

### 5.1 Targeted policy interventions

The persistent gap between regions, particularly the lower outcomes in Africa and South-East Asia compared to the Americas and Eastern Mediterranean, indicates that global averages are insufficient for policymaking. Governments and international organizations should design context-specific programs that address local barriers to healthcare access and delivery.

### 5.2 Resource allocation and prioritization

Forecasting results show divergent future trajectories depending on the model applied. This suggests the need for proactive allocation of financial and human resources in anticipation of both conservative and accelerated growth scenarios. Countries predicted to stagnate or decline under certain models should be prioritized for capacity-building and investment.

### 5.3 Integration of sex-disaggregated monitoring

The consistent higher values and greater variability observed among females highlight the need for continued collection and analysis of sex-disaggregated data. This ensures that inequities are not masked in aggregated indicators and that programs are sensitive to the unique needs of men and women.

### 5.4 Forecasting for strategic planning

The application of machine learning demonstrates that health forecasting is inherently uncertain and model-dependent. Rather than relying on a single predictive outcome, policymakers should adopt multi-model or ensemble approaches to better prepare for a range of plausible futures. This strengthens resilience in health systems and supports adaptive strategies.

### 5.5 Advancing research and collaboration

Finally, the integration of statistical and machine learning methods in this study demonstrates the value of interdisciplinary approaches. Future research should expand by including socioeconomic, environmental, and behavioral covariates to refine predictions. Collaborative networks that combine epidemiological expertise, data science, and policy planning can ensure findings are translated into impactful real-world actions.

## 6 Conclusion

Using statistical analysis, visualization, and machine learning forecasting, this study investigated differences and temporal trends in the measured indicator across gender and global areas. The results consistently showed sex-based differences with women displaying greater variability and higher values than men over nearly all visualizations. These disparities were consistent over time, pointing to structural or biological effects that endure despite more general worldwide developments.

Regional studies drew attention to major geographic disparities, with Africa and South-East Asia always grouping at lower numbers whereas the Americas and Eastern Mediterranean showed higher and more variable results. Europe and the Western Pacific sat between positions yet displayed interior diversity. These findings demonstrate that global averages can obscure substantial national and regional discrepancies, underscoring the need for context-specific policy measures and interventions.

Time-series and regression studies showed steady upward curves in most groups, indicating a gradual worsening in insufficient physical activity over time but at varying speeds depending on sex and location. Machine learning predicts possible future outcomes, therefore building on this study. While neural (MLP) and kernel-based models (SVR) showed sustained or accelerated expansion, tree-based models (XGBoost, Random Forest) predicted conservative or falling trajectories. This difference emphasizes the need of ensemble methods and multi-model forecasting to strike a balance between near-term stability and long-term credibility.

By using modern machine learning techniques in conjunction with classical statistical approaches, this paper offers a thorough, data-driven perspective on inequities and future paths. The results underscore the need to consider biological, social, and regional contexts when interpreting findings and emphasize the importance of predictive modeling for proactive policy and planning. Future research should build on these observations by adding extra covariates, improving model ensembles, and matching projections to actual policy frameworks to increase resiliency against uncertainty. Strengths of this study include the use of a large, publicly available longitudinal WHO dataset spanning 23 years and 195 countries, the application of four complementary ML models, sex-disaggregated analysis enabling gender-specific inference, and a fully reproducible Python-based analysis pipeline. Limitations include reliance on country-level aggregate data without individual-level covariates, the inherent extrapolation constraints of tree-based models, and the absence of data on policy interventions or socioeconomic changes that may influence future physical activity trends. These limitations should be addressed in future studies through the inclusion of additional determinants and prospective validation of forecasts against emerging surveillance data.

## Data Availability

The raw data supporting the conclusions of this article will be made available by the authors, without undue reservation.
